# Supplementary material for: The efficiency of migration and profile control with emulsion systems in class III reservoirs
Source: R Soc Open Sci. 2019 May 22;6(5):181634. doi: 10.1098/rsos.181634 (PMC6549995; doi:10.1098/rsos.181634)
Supplement: all figures [file rsos181634supp1.docx]

**Figures：**

1-Plunger pump; 2-Beaker; 3-Middle container; 4-Pressure gange; 5-Core holder; 6-Graduated cylinder; 7-Manual pump; 8-Oven

Fig.1 Experimental-process diagram


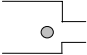

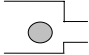

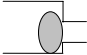


(a) (b) (c)

Fig.2 Matching relationship between the droplets of emulsion and the diameter of the pore throat

Fig.3 Viscosity of emulsions under different surfactant concentrations

Fig.4 F_R_ when emulsions with different water cut flow through 20mD core

Fig.5 F_R_ when emulsions with different water cut flow through 40mD core

Fig.6 F_R_ when emulsions with different water cut flow through 60mD core

Fig.7 F_R_ when emulsions with different water cut flow through 80mD core

Fig.8 F_R_ when emulsions with different surfactant concentrations flow through 20mD core

Fig.9 F_R_ when emulsions with different surfactant concentrations flow through 40mD core

Fig.10 F_R_ when emulsions with different surfactant concentrations flow through 60mD core

Fig.11 F_R_ when emulsions with different surfactant concentrations flow through 80mD core


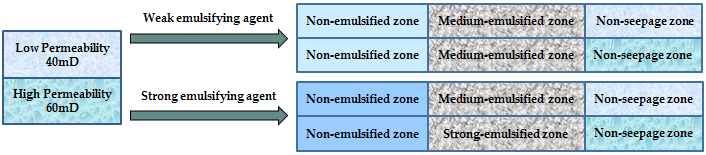


Fig.12 Profile control enficacy when the permeability contrast is 1.5


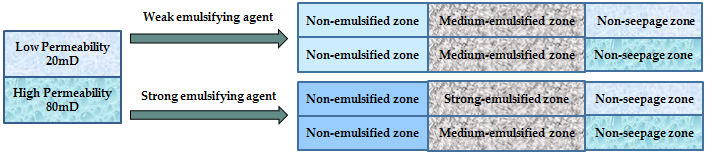


Fig.13 Profile control enficacy when the permeability contrast is 4
