# Supplementary material for: The efficiency of migration and profile control with emulsion systems in class III reservoirs
Source: R Soc Open Sci. 2019 May 22;6(5):181634. doi: 10.1098/rsos.181634 (PMC6549995; doi:10.1098/rsos.181634)
Supplement: all tables [file rsos181634supp2.docx]

**Tables：**

Table.1 Formula of prepared brine

| Salinity  (mg/L) | Sodium chloride  (g/L) | Potassium chloride  (g/L) | Calcium chloride  (g/L) | Magnesium sulphate  (g/L) | Sodium sulphate  (g/L) | Sodium bicarbonate  (g/L) |
| --- | --- | --- | --- | --- | --- | --- |
| 6778 | 3.489 | 0.020 | 0.064 | 0.262 | 0.114 | 2.829 |

Table.2 Basic core parameters under different water cut

| Pore diameter  (µm) | Water cut  (%) | Diameter of emulsion  (µm) | De/Dp | Outlet size of emulsion  (µm) |
| --- | --- | --- | --- | --- |
| 1.804  (20mD) | 30 | 1.136 | 0.63 | 1.129 |
|  | 40 | 1.975 | 1.09 | 1.764 |
|  | 50 | 2.891 | 1.60 | 1.054 |
|  | 60 | 2.131 | 1.18 | 1.332 |
|  | 70 | 1.342 | 0.74 | 1.020 |
| 3.126  (40mD) | 30 | 1.136 | 0.36 | 1.131 |
|  | 40 | 1.975 | 0.63 | 1.753 |
|  | 50 | 2.891 | 0.92 | 2.656 |
|  | 60 | 2.131 | 0.68 | 2.032 |
|  | 70 | 1.342 | 0.43 | 1.232 |
| 4.615  (60mD) | 30 | 1.136 | 0.25 | 1.116 |
|  | 40 | 1.975 | 0.43 | 1.664 |
|  | 50 | 2.891 | 0.63 | 2.447 |
|  | 60 | 2.131 | 0.46 | 2.003 |
|  | 70 | 1.342 | 0.29 | 1.035 |
| 5.923  (80mD) | 30 | 1.136 | 0.19 | 1.129 |
|  | 40 | 1.975 | 0.33 | 1.578 |
|  | 50 | 2.891 | 0.49 | 2.479 |
|  | 60 | 2.131 | 0.36 | 1.912 |
|  | 70 | 1.342 | 0.23 | 1.165 |

Table.3 Basic core parameters under different surfactant concentration

| Pore diameter  (µm) | Surfactant concentration  (%) | Diameter of emulsion  (µm) | De/Dp | Outlet size of emulsion  (µm) |
| --- | --- | --- | --- | --- |
| 1.804  (20mD) | 1 | 2.891 | 1.60 | 1.129 |
|  | 1.5 | 2.437 | 1.35 | 1.764 |
|  | 2 | 1.963 | 1.09 | 1.054 |
| 3.126  (40mD) | 1 | 2.891 | 0.93 | 2.621 |
|  | 1.5 | 2.437 | 0.78 | 2.142 |
|  | 2 | 1.963 | 0.63 | 1.752 |
| 4.615  (60mD) | 1 | 2.891 | 0.63 | 2.634 |
|  | 1.5 | 2.437 | 0.53 | 2.042 |
|  | 2 | 1.963 | 0.43 | 1.664 |
| 5.923  (80mD) | 1 | 2.891 | 0.49 | 2.631 |
|  | 1.5 | 2.437 | 0.41 | 1.942 |
|  | 2 | 1.963 | 0.33 | 1.578 |

Table.4 Emulsion grading plate

| Permeability（mD） | Surfactant concentration  (%) | Water cut（%） | | | | |
| --- | --- | --- | --- | --- | --- | --- |
|  |  | 30 | 40 | 50 | 60 | 70 |
| 20 | 1 | □ | △ | △ | □ | ○ |
|  | 1.5 | □ | △ | △ | □ | ○ |
|  | 2 | △ | △ | △ | △ | □ |
| 40 | 1 | ○ | □ | △ | □ | ○ |
|  | 1.5 | □ | □ | △ | □ | ○ |
|  | 2 | △ | △ | △ | △ | □ |
| 60 | 1 | ○ | ○ | □ | □ | ○ |
|  | 1.5 | □ | □ | △ | □ | ○ |
|  | 2 | □ | △ | △ | △ | □ |
| 80 | 1 | ○ | ○ | □ | □ | ○ |
|  | 1.5 | ○ | □ | △ | □ | ○ |
|  | 2 | □ | □ | △ | □ | ○ |
